# The synergistic effect of grafting and LED light quality on enhancing the mineral nutrition and growth performance of tomato seedlings

**DOI:** 10.1038/s41598-026-38960-3

**Published:** 2026-02-12

**Authors:** Seyedreza Soltani, Hossein Aroiee, Reza Salehi, Nazim S. Gruda

**Affiliations:** 1 Department of Horticultural Sciences, Ferdowsi University of Mashhad, Mashhad, Iran; 2 Department of Horticultural Sciences, Faculty of Agriculture, Ferdowsi University of Mashhad, Mashhad, Iran; 3 Department of Horticultural Sciences, Campus of Agriculture and Natural Resources, University of Tehran, Karaj, Iran; 4 Department of Horticultural Science, INRES–Institute of Crop Science and Resource Conservation, University of Bonn, Bonn, Germany

**Keywords:** Controlled environment agriculture, CEA, Rootstock, Transplant production, Photomorphogenesis, Physiology, Plant sciences

## Abstract

Light quality is a critical determinant in controlled environment agriculture, yet information regarding the interactive effects of spectral composition and grafting on tomato seedling performance remains limited. Moving beyond the assumption that rootstock vigor is solely a static trait, we hypothesized that above-ground spectral cues significantly modulate rootstock efficiency. We investigated the synergistic effects of grafting (*Solanum lycopersicum* ‘Maxifort’) and various LED spectra (monochromatic Red, Blue, White, and Red:Blue 70:30) on morphological architecture, photosynthetic potential, and the stoichiometric balance of mineral nutrients in tomato seedlings. Our results reveal a critical interaction: while grafting alone alleviated specific nutritional deficits (N, K, Mg) under suboptimal monochromatic red light, the rootstock’s capacity to maximize the uptake of key elements—particularly phosphorus and calcium—was fully realized only under the synergistic Red:Blue spectrum. This study provides empirical evidence that integrating the R70:B30 spectrum with grafting not only improves growth but also optimizes resource acquisition. These findings offer a novel approach to optimizing transplant quality and establish a robust protocol for producing resilient transplants in modern nurseries. Future research should focus on unraveling the molecular pathways underlying this light-rootstock communication.

## Introduction

Tomato (*Solanum lycopersicum* L.) is widely recognized as one of the most economically important vegetable crops globally. It is considered the second most important vegetable in the world, only after the potato, with an annual global production exceeding 190 million tonnes. This crop serves as a vital source of income for growers. It plays a crucial role in ensuring global nutritional security, due to its dual utility for both fresh consumption and industrial processing^1,2^. Beyond its economic value, the tomato is considered a functional food due to its rich nutritional profile, containing essential vitamins, minerals, and bioactive compounds, particularly lycopene^3^. Epidemiological studies have consistently linked regular tomato consumption to a reduced risk of chronic diseases, including cardiovascular disorders and certain cancers, mainly due to its potent antioxidant properties^4^. From an economic perspective, the commercial production of grafted vegetable transplants has evolved into a large-scale, specialized global industry, driven by the phasing out of chemical soil fumigants and by intensifying demand for sustainable yield. Currently, the adoption rate of grafted seedlings for solanaceous crops in high-tech greenhouses has reached nearly 90–100% in countries such as Japan, Korea, and the Netherlands. Despite the higher cost of grafted seedlings—typically 2 to 3 times that of non-grafted seedlings—their use is expanding globally due to the significant return on investment (ROI) from improved yields and reduced reliance on agrochemicals^5–7^.

Furthermore, tomato serves as a prime model organism for physiological research, making it an excellent candidate for investigating the synergistic effects of grafting and environmental cues such as light quality. Horticulturists have long used grafting to boost the productivity and resilience of vegetables. It is a key strategy for overcoming agricultural challenges, such as soil-borne pathogens, nematode infestations, and harsh environmental conditions, by enhancing plant vigor, yield, and quality^8,9^. Grafting also improves water-use efficiency and increases resistance to salinity and flooding^10–12^. Beyond yield improvement and stress tolerance, grafting has been identified as a sustainable agronomic strategy to enhance the nutritional profile of vegetables, specifically by modifying ion uptake and increasing the concentration of bioactive compounds in the scion^13^. A recent global analysis of vegetable transplant research indicates that applying technologies such as grafting and LED lighting during the seedling stage is a rapidly expanding strategy to enhance not only production efficiency but also the sustainable nutritional quality of the final produce^5^. Consequently, successful grafting involves a complex interplay of hormonal signals, especially auxin and cytokinin. It is heavily influenced by environmental factors such as light^14^, which not only drives photosynthesis but also controls plant growth and development^15^.

The spectral composition, photoperiod, and intensity of light affect plant metabolism and interact with other environmental variables, such as temperature, humidity, and CO_2_ concentration, to influence overall plant behavior^16,17^. Furthermore, recent reviews highlight that directionality and energy of light are also crucial determinants regulating plant morphogenesis and assimilate partitioning^18,19^. Light’s function is thus twofold: acting as the energy source for carbon fixation and the informational signal for morphogenesis. These specific light signals are decoded by a complex network of photoreceptors, including phytochromes, cryptochromes, and phototropins, triggering adaptive physiological responses^19^. For greenhouse-grown crops, artificial lighting is often used to ensure sufficient light energy^20^. Low light intensity and short photoperiods, which are commonly limiting factors in winter, can reduce tomato yield and quality^21^. Although increasing yields through optimized grow lights and elevated CO_2_ is well established^22^, recent approaches no longer focus solely on maximizing production. Instead, supplemental lighting is now used to manipulate pre-harvest conditions to improve product quality^13^. For instance, precise spectral control using LEDs enables stage-specific metabolic regulation, such as applying a higher blue-to-red ratio for seedlings or supplemental UV-A during fruiting stages to boost phytochemical accumulation^13,18^.

Using LED devices solves several problems inherent in traditional lighting. Unlike conventional light sources like high-pressure sodium (HPS) or metal halide (MH) lamps, which have fixed brightness and broad spectra, LEDs offer flexible, adjustable spectral outputs. Additionally, LEDs provide focused, efficient lighting and are more environmentally friendly^23^. Indeed, this technology has become increasingly significant not only for its capability to deliver specific light spectra that promote plant growth but also for its superior energy efficiency, which can reduce electricity usage by 20–92% compared to traditional systems^24^. Red and blue wavelengths are the most common spectra used to control light quality and influence plant growth. These light qualities affect a wide range of plant responses, ranging from photomorphogenesis to photosynthetic efficiency^25,26^. In tomato cultivation, these spectra regulate stomatal conductance and the synthesis of essential pigments, such as chlorophylls and carotenoids, ultimately enhancing yield^27^. While red light primarily drives reproductive growth and chloroplast function, blue light mediates photomorphogenic responses, including leaf expansion, stomatal regulation, and pigment accumulation^28,29^. Specifically, regarding plant architecture, blue light is known to regulate plant growth, primarily by preventing excessive stem elongation. Red light, on the other hand, promotes the elongation of tomato petioles and stems, leading to a less compact plant structure^30^. However, relying solely on monochromatic red or blue light is often insufficient for full development; therefore, adjusting the red-to-blue (R/B) ratio is essential to optimize vegetative and reproductive growth^21^. Previous reports have shown that the biomass and shape of plants, such as cucumber, tomato, and lettuce, are significantly influenced by the combination of red and blue light^31–33^.

Recent comprehensive reviews identify grafting and LED lighting as the most rapidly advancing practices in vegetable seedling production, crucial for enhancing efficiency and sustainability in modern nurseries^34^. However, despite the crucial role of light quality in vegetable crop performance, the physiological mechanisms through which spectral composition influences rootstock efficiency remain largely unexplored. While most researchers have focused on the individual effects of light or grafting, a significant gap remains in understanding how above-ground light signaling regulates nutrient uptake in seedling roots. Based on this, we hypothesized that the nutritional advantage of a vigorous rootstock is not solely an inherent trait but depends on specific spectral cues to be fully activated. Furthermore, we postulated that grafting could compensate for the growth limitations and nutrient imbalances typically associated with suboptimal monochromatic lighting. To address these hypotheses, the present study aims to decipher the complex interaction between different LED light spectra and grafting status on the growth parameters and mineral nutrient stoichiometry of tomato seedlings. Notably, this research aims to clarify the mechanisms underlying light-driven nutrient selectivity. The findings are expected to provide new physiological insights for optimizing nursery production, demonstrating how combining specific light ratios with grafting can enhance seedling quality by improving resource utilization.

## Results

### Growth parameters and SPAD value

**Fig. 1:**
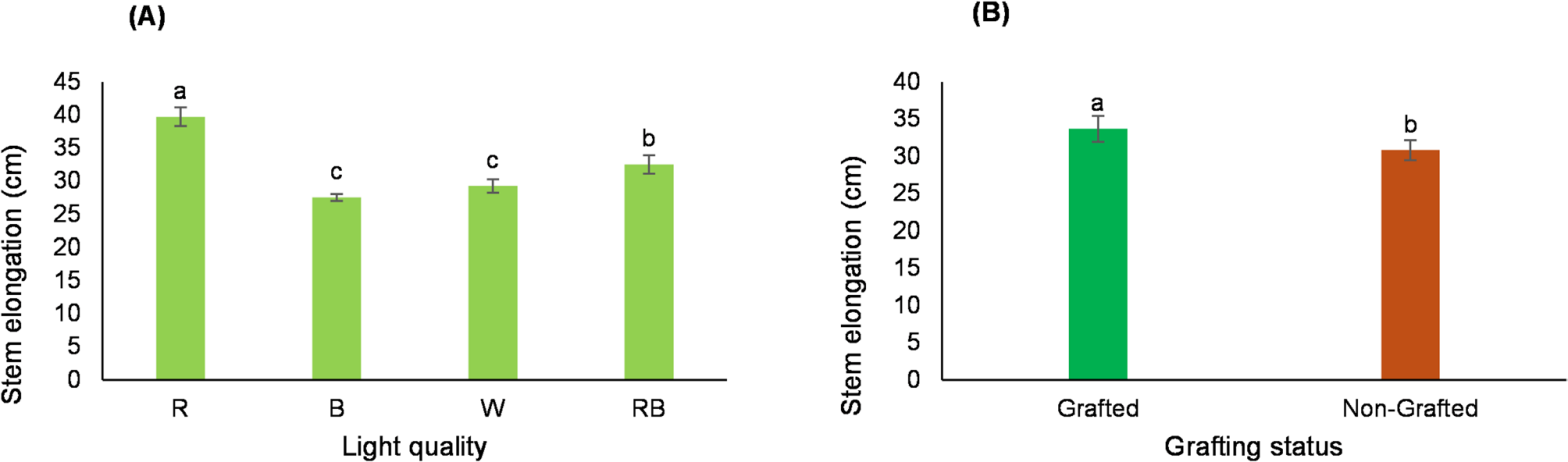
Main effects of different light treatments (**A**) and grafting (**B**) on stem elongation of tomato seedlings. Data represent the mean ± s.e.m. (*n* = 3). ANOVA results indicated significant main effects for light quality (*P* < 0.0001) and grafting (*P* = 0.0029). Different letters above the bars within each chart indicate significant differences according to the LSD test (*p* ≤ 0.05).

Analysis of variance revealed that light quality and grafting had significant independent effects on seedling stem elongation. Mean comparisons revealed that the tallest and shortest stems were observed in seedlings exposed to red and blue light, respectively. Although blue light produced the shortest stems, this difference was not statistically significant compared with seedlings grown under W light (Fig. 1A). The pronounced stem elongation under R light is consistent with previous findings^35–37^, which attributes this response primarily to phytochrome-mediated internode elongation rather than an increase in node number. Additionally, the results demonstrated that stem elongation was greater in grafted seedlings than in non-grafted ones (Fig. 1B).

**Fig. 2:**
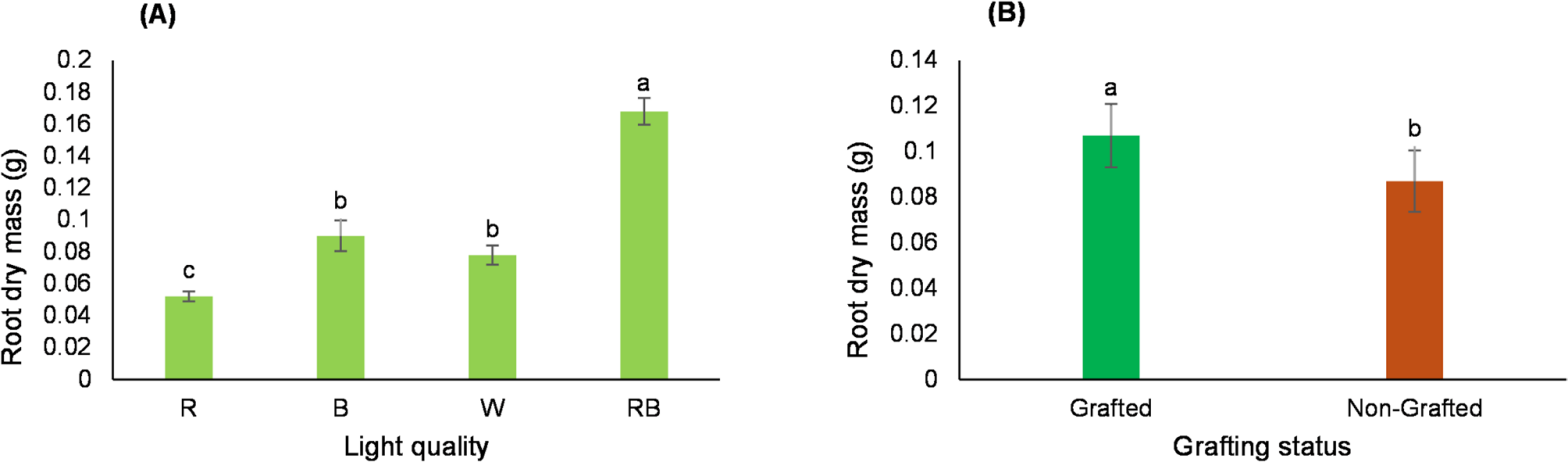
Main effects of different light treatments (**A**) and grafting (**B**) on root dry mass of tomato seedlings. Data represent the mean ± s.e.m. (*n* = 3). ANOVA results indicated significant main effects for light quality (*P* < 0.0001) and grafting (*P* = 0.0065). Different letters above the bars within each chart indicate significant differences according to the LSD test (*p* ≤ 0.05).

The results revealed that root dry mass was significantly influenced by both light quality and grafting. Regarding light quality, the highest root dry mass was observed in seedlings exposed to the R:B light treatment (Fig. 2A). As for the grafting effect, root dry mass was greater in grafted seedlings than in non-grafted seedlings (Fig. 2B).

According to the results, a significant interaction between light quality and grafting was observed for total dry mass, leaf area, and SPAD value. Mean comparisons indicated that the highest total dry mass, leaf area, and SPAD value were observed in grafted seedlings exposed to the R:B light treatment. Additionally, the lowest total dry mass and leaf area were observed in non-grafted seedlings exposed to the R light treatment. However, for the SPAD value, while the lowest readings were also recorded under R light, no significant difference was observed between grafted and non-grafted seedlings (Table 1).

**Table 1:** Effect of light quality and grafting on growth characteristics and SPAD value of grafted and non-grafted (Non-G) tomato seedlings. Data represent the mean ± s.e.m. (*n* = 3). Different letters in each column indicate significant differences according to the LSD test (*p* ≤ 0.05).

Traits	Total dry mass (g)		Leaf area (cm^2^)	SPAD value
Red	Grafted	0.84 ± 0.02^e^	482.04 ± 19.17^d^	34.39 ± 0.31^e^
	Non-G	0.68 ± 0.01^f^	368.04 ± 9.00^e^	33.52 ± 0.52^e^
Blue	Grafted	1.24 ± 0.02^c^	585.41 ± 9.06^c^	39.83 ± 0.59^c^
	Non-G	0.97 ± 0.01^d^	557.46 ± 7.10^c^	36.65 ± 0.90^d^
White	Grafted	1.00 ± 0.02^d^	567.12 ± 14.11^c^	35.16 ± 0.87^de^
	Non-G	0.84 ± 0.02^e^	465.12 ± 6.32^d^	38.50 ± 0.30^c^
Red:Blue (70% R)	Grafted	1.74 ± 0.01^a^	729.16 ± 23.22^a^	44.13 ± 0.40^a^
	Non-G	1.61 ± 0.01^b^	630.35 ± 5.48^b^	42.22 ± 0.23^b^
*P*	–	0.0024	0.0156	0.0003

### Total mineral nutrient content

**Fig. 3:**
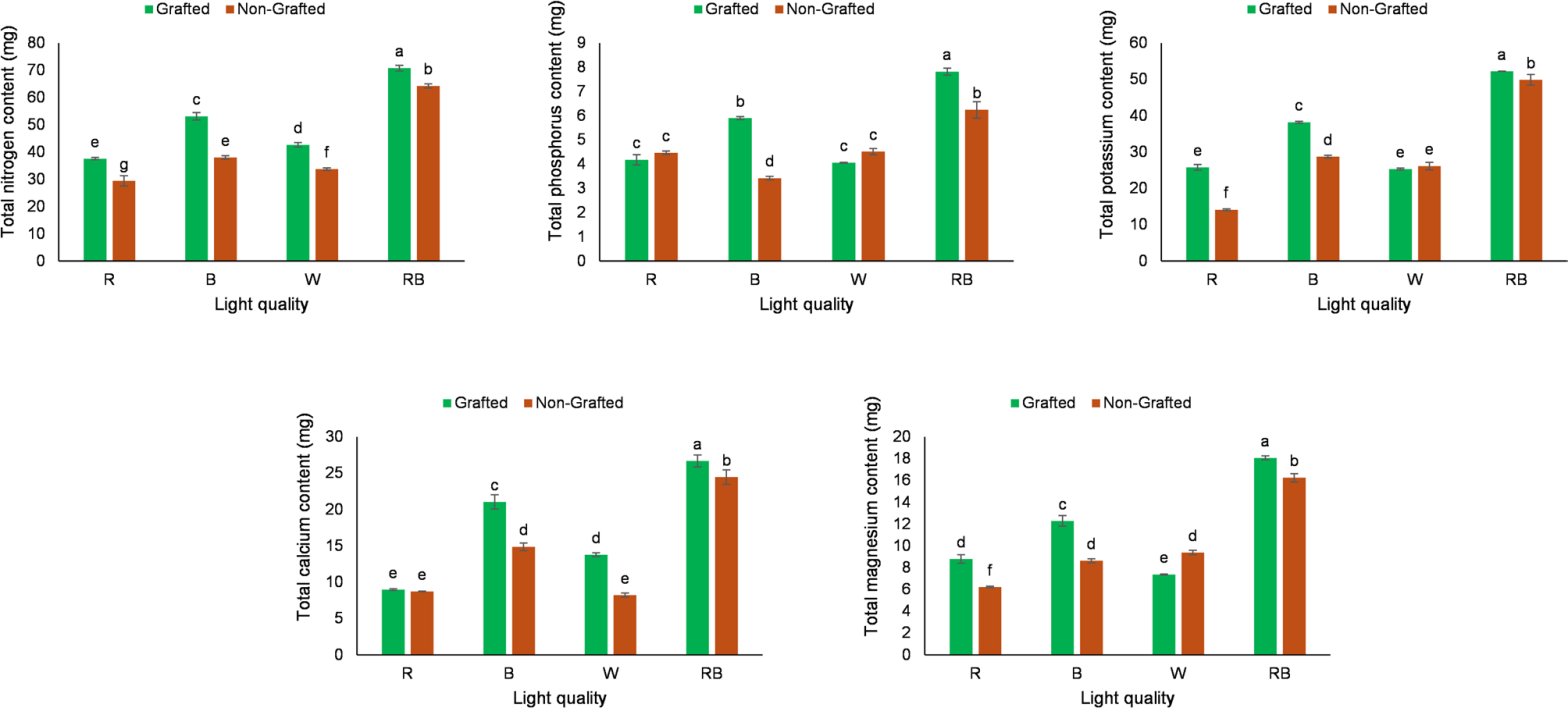
Interactive effects of light quality and grafting on the total content of nitrogen, phosphorus, potassium, calcium, and magnesium in grafted and non-grafted tomato seedlings. Data represent the mean ± s.e.m. (*n* = 3). ANOVA results indicated significant Light quality × Grafting interactions for N (*P* = 0.0087), P (*P* < 0.0001), K (*P* < 0.0001), Ca (*P* = 0.0004), and Mg (*P* < 0.0001). Different letters above the bars within each chart indicate significant differences according to the LSD test (*p* ≤ 0.05).

The interactive effect of light quality and grafting significantly affected the total content of all five elements. According to the results, the highest total content for all five elements was observed in grafted tomato seedlings exposed to a R:B light treatment. In contrast, the lowest nutrient accumulation—particularly for nitrogen, potassium, and magnesium—was generally recorded in non-grafted seedlings under monochromatic R light. Furthermore, comparing light quality revealed that the R:B treatment consistently resulted in higher mineral content than W light, B light, or R light alone. Interestingly, grafting alleviated the nutritional deficit in seedlings grown under suboptimal light conditions, as grafted plants consistently showed higher elemental concentrations than non-grafted plants across most light treatments. However, in some specific cases, no significant difference was observed between grafted and non-grafted seedlings. For instance, the phosphorus and calcium content under R light treatment, as well as the phosphorus and potassium levels under W light treatment, did not differ significantly (Fig. 3).

**Fig. 4:**
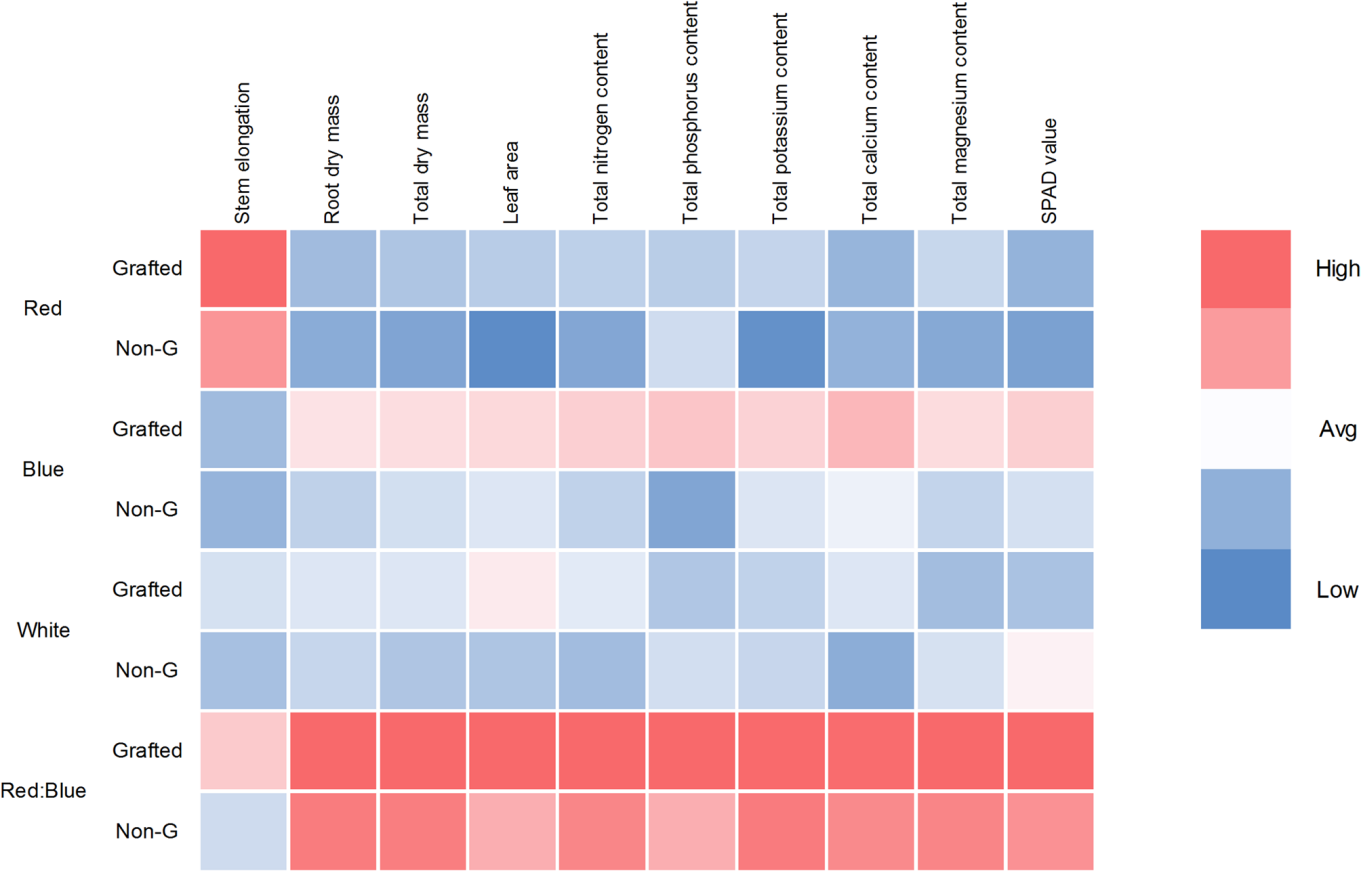
Heatmap visualization of the interactive effects of light quality and grafting on morphological traits, nutrient content, and SPAD value of grafted and non-grafted (Non-G) tomato seedlings. The color scale represents each parameter’s relative performance, from low (blue) to high (red).

To visualize the comprehensive interaction between grafting and light quality, a heatmap analysis was performed using standardized values (Z-scores) for all measured parameters (Fig. 4). This visualization enables direct comparison of traits with different measurement units. As indicated by the color intensity, where red represents values above the mean (high Z-scores) and blue indicates values below the mean (low Z-scores), the combination of grafting with the R:B light spectrum resulted in the highest values across most morphological and nutritional traits. In contrast, non-grafted seedlings under monochromatic lights (especially R) exhibited the lowest values.

## Discussion

Grafted tomato seedlings, when exposed to R light, exhibited the greatest stem elongation^38^. Similarly, grafted watermelon seedlings when exposed to R light had the longest stems, whereas seedlings under B or R:B mixed light (36% B) had shorter stems^39^. Cucumber seedlings exposed to monochromatic R light exhibited greater plant height than those that received R:B light (70R30B treatment)^26^. Moreover, the ‘Rejina’ tomato cultivar showed greater plant height under R light compared to W light^40^. Comparable results have been reported in other species, including chrysanthemum, lettuce, and coriander, where R light promotes stem elongation and B light inhibits it^33,41,42^. The reduction in stem length under B light is primarily due to photoreceptor activity. Signals generated by cryptochromes and phototropins (blue light-sensitive receptors) are known to inhibit stem elongation, while phytochromes (red light-sensitive receptors) promote cell division and elongation. Consequently, stem length typically decreases under B light and increases under R light^43–47^. Additionally, these changes are associated with gibberellin (GA) biosynthesis, which accelerates stem elongation. Red light enhances GA content, thereby promoting stem growth, whereas B light reduces GA levels. Blue light also increases indole-3-acetic acid (IAA) levels, which modulate genes controlling GA biosynthesis, thereby reducing stem elongation^46,48–51^. Furthermore, the significant impact of light quality on grafted seedlings observed in our study can be explained by its role in hormonal regulation and tissue regeneration, which are essential processes for successful graft union formation and subsequent growth^34^. It has been reported that B light reduced both stem length and internode distance compared with R light, and that supplementation with B light shortened internodes in tomato^35,36^. Similarly, pennyroyal plants exposed to monochromatic R light exhibited greater internode length than those exposed to B, W, or R:B (3:1) light combination^37^.

Grafted tomato seedlings grown under a R:B light combination (B30%) exhibited the largest leaf area^38^. Similarly, cucumber seedlings exposed to the 9R1B light treatment had greater leaf area than those cultivated under monochromatic R, B, or W light^26^. Furthermore, soybean seedlings cultivated under various R:B light combinations showed greater leaf area in comparison with seedlings grown under R light^45^. In another study, grafted watermelon seedlings exposed to a combination of R and B light (B32%) had larger leaf area than those exposed only to R light^52^. A 3:1 mix of R and B light resulted in an increased leaf area in *Sedum rubrotinctum* plantlets when compared to using monochromatic R and B light^53^. Consistently, grafted watermelon seedlings exposed to a combination of R and B light (B24%) were observed to have greater leaf area compared to seedlings under R light^39^. All these findings suggest a synergistic balance between phytochrome- and cryptochrome-mediated responses under combined spectra. While red light promotes cell expansion, the concurrent presence of blue light is essential to regulate this expansion, preventing excessive etiolation while promoting proper leaf blade development and stomatal density. This results in an optimized light interception capacity without the structural weakness associated with shade-avoidance responses often triggered by monochromatic red light^42,45,54^.

In a similar study, R:B light (R70:B30%) yielded greater root dry mass compared to seedlings cultivated beneath either monochromatic R or B light^38^. Other studies have shown that pepper and kale plants exposed to different R:B light ratios exhibited higher total fresh and dry mass than those exposed to monochromatic R light^33^. Similarly, in a study on coriander, key leaf parameters, including dry mass and area, of plants exposed to R:B light (R87:B13%) were higher than those of plants exposed to B light^41^. Soybean seedlings exposed to different R:B light ratios also had greater root and total dry mass than seedlings exposed to R light^45^. Additionally, the total dry mass reached its highest value under the R:B light (9R1B) treatment. In comparison, under monochromatic R light, these parameters were significantly reduced and were also lower under monochromatic B and W light^26^. Furthermore, in a study on greenhouse tomato plants, shoot dry mass in plants grown under different R:B light combinations (R92:B8%)(R96:B4%) was greater than in tomato plants grown under monochromatic R light^36^. Results from grafting tomatoes onto three commercial rootstocks showed that the highest shoot dry mass was obtained with ‘Maxifort’ and ‘Unifort’^55^. In another similar study on tomato seedlings, after 30 days, root and shoot dry mass in seedlings exposed to combined R and B light (R75:B25%) were greater than in seedlings exposed to W and B light treatments, with the lowest root and shoot dry mass being obtained in seedlings grown under R light^51^. Consistent with our findings, recent comprehensive reviews emphasize that tailored lighting strategies, specifically combining R and B wavelengths, are crucial for optimizing seedling development and biomass accumulation in controlled environments^13^. Moreover, the superior performance of grafted seedlings under this combined R:B spectrum in our study is consistent with global research trends, which identify this specific spectral combination as highly effective for promoting plant dry weight and photosynthetic activity in vegetable nurseries^5^.

Mechanistically, the superior biomass accumulation under the R:B spectrum suggests that the inclusion of blue light mitigates ‘red light syndrome’—characterized by biochemical dysfunction and reduced stomatal conductance—thereby optimizing photosynthetic efficiency^52,54^. Furthermore, blue light signals are crucial for the export of photosynthates (sucrose) from source leaves to sink organs, which explains the observed maximization of root dry mass^29^. Practically, this efficient assimilate partitioning results in a robust root system, which is the primary indicator of high-quality transplants. For growers, this implies that spectral tuning acts as a non-chemical growth regulator, producing seedlings with superior hydraulic conductivity and stress resilience^56,57^.

In the present study, the highest total content of all five elements (mg) was observed in seedlings under the R:B light treatment. These findings align with the results of other researchers, despite differences in measurement metrics. For example, in a study on coriander, the highest leaf Phosphorus concentration was also obtained under R:B light (R87:B13%), while the lowest concentration was observed under R light^41^. This convergence indicates that the synergistic combination of R and B light is highly effective for element uptake, whether assessed by tissue concentration or total plant accumulation. In a study on mustard microgreens, the combined application of R and B lights improved mineral absorption^58^. Other studies have also found that combining B light with R light can improve magnesium absorption^59^. We observed in this study that the maximum element uptake occurred in grafted seedlings under an R:B light ratio.

We propose that two key light-dependent mechanisms drive this enhanced nutrient profile. First, blue light stimulates stomatal opening via phototropins, increasing the transpiration stream that facilitates the mass flow of immobile nutrients like Calcium and Magnesium^42,58^. Second, the specific spectral quality regulates membrane transport systems, generating the necessary gradient for the active uptake of ions such as Nitrogen and Potassium^57^. This indicates that the nutritional advantage of vigorous rootstocks like ‘Maxifort’ is not static but requires specific spectral cues—specifically a balanced R:B ratio—to unlock their ion-uptake potential fully.

In a study on strawberry plants, R and B light wavelengths affect mineral element absorption and photosynthetic performance, thereby increasing plant resistance to salinity and alkaline stresses^56^. In another study, the effect of grafting tomato onto three rootstocks (‘Beaufort’, ‘He-Man’, and ‘Resistar’) was evaluated, and it was found that the highest calcium concentration was in the leaves of grafted tomatoes, whereas no difference was found in leaf potassium concentration between grafted and non-grafted tomatoes^60^. Our findings indicate that the efficacy of grafting in enhancing mineral uptake is strongly modulated by light quality. While grafting generally promoted nutrient accumulation, this advantage was not uniform across all spectral conditions. Specifically, the rootstock-mediated improvement in nutrient acquisition was limited for some aspects under monochromatic R and W light. For instance, no significant grafting benefit was observed for phosphorus and calcium under R light, nor for phosphorus and potassium under W light, suggesting that suboptimal light spectra may constrain the root system’s potential to take up specific ions. It has been reported that the mineral element accumulation in the leaves of grafted plants depends on the rootstock and scion combination. Therefore, using a suitable rootstock can have a positive impact on the mineral content in the shoot of grafted seedlings and, consequently, their better growth^61^.

The enhanced nutrient uptake observed under the R:B spectrum aligns with recent insights suggesting that the light environment influences mineral balance in a complex way, with specific wavelengths having distinct functions in modulating the physiological mechanisms of nutrient acquisition and plant development^13^. Blue light, for example, opens ion channels, thereby increasing the plant’s mineral content. Red light, conversely, is reported to enhance the root system’s capacity for water and mineral absorption, thereby facilitating the transport and utilization of these nutrients^57,62^. Increasing the B light percentage from 50 to 100% in Brassicaceae microgreens typically resulted in higher concentrations of phosphorus (P), calcium (Ca), and magnesium (Mg). In this study, the highest potassium (K) concentration (in mustard) was observed under 100% B light, while nitrogen (N) was excluded from this positive trend. Two possible reasons were suggested for this phenomenon: first, that B light opens ion channels in the cell membrane via phototropins and enhances ion transport, and second, that higher proportions of B light improve the “Translocation factor” (TF), aiding in the more effective transfer of elements from the roots to the shoots^63^. In addition to its effect on membrane transport, the B light present in the combined spectrum plays a key role in regulating stomatal opening and increasing transpiration^42^. This process serves as the primary driver of the uptake of mass-flow-dependent nutrients, such as calcium. Consequently, this mechanism likely accounts for the maximized calcium accumulation observed under the RB treatment in our study, as well as the superior calcium uptake in seedlings exposed to monochromatic B light compared to those under red light, where stomatal conductance is typically impaired. It has also been reported that LED light at appropriate wavelengths can stimulate the secretion of phytohormones, particularly gibberellins, thereby enhancing the uptake of mineral elements and improving plant growth^64^.

In the current study, the highest SPAD values were recorded under the combined R:B light, whereas monochromatic R light resulted in the lowest chlorophyll index. These findings are consistent with previous reports indicating that relative chlorophyll content was generally higher in plants grown under R:B combinations compared to the W light control^65^. Similarly, the reduction of SPAD values under monochromatic R light in our experiment aligns with observations in watermelon scion leaves, where R light significantly decreased chlorophyll content^52^. This reduction in chlorophyll content and weaker physiological performance under monochromatic R light can be attributed to the ‘red light syndrome.’ This condition is characterized by impaired stomatal function, reduced chlorophyll biosynthesis, and structural abnormalities in chloroplasts, necessitating the addition of a percentage of B light to alleviate these disorders^36,52,54^. The enhanced chlorophyll accumulation observed under the mixed spectrum (RB) in our study could be attributed to specific molecular regulations involving photoreceptor activation. It has been demonstrated that complex spectra are perceived by photoreceptors (SlPHYB1 and SlCRY1), which activate the transcription factor SlHY5. SlHY5, in turn, directly promotes the expression of chlorophyll synthesis genes (*SlLHCA*, *SlLHCB*, and *SlCYCB*), thereby leading to higher chlorophyll content compared to monochromatic treatments^66^.

## Conclusions

This study confirms that relying on monochromatic lighting, specifically R or B alone, is insufficient for optimizing the growth and quality of tomato seedlings in controlled environments. Instead, our findings demonstrate the clear superiority of the combined R:B light spectrum (R70:B30). This treatment created a synergistic effect that not only mitigated the morphological limitations associated with monochromatic lights (such as excessive elongation under R light) but also maximized photosynthetic potential and significantly enhanced the total accumulation of key macronutrients (N, P, K, Ca, and Mg). Furthermore, this research highlights the efficacy of grafting onto ‘Maxifort’ rootstock as a powerful strategy for improving seedling vigor. Importantly, our results revealed a dynamic interaction: while grafting alleviated nutritional deficits under suboptimal light conditions, its capacity to maximize nutrient uptake was fully realized only under the optimized R:B spectrum. Consequently, integrating grafting with a 70:30 red-to-blue ratio offers a robust protocol for producing high-quality transplants in commercial nurseries. From a broader perspective, while this study challenges the assumption that rootstock efficiency is solely a static trait, it primarily establishes the physiological basis for light-mediated rootstock programming. While this study successfully demonstrates the phenotypic and nutritional benefits of integrating grafting with the R:B spectrum, we acknowledge that the underlying molecular mechanisms remain to be fully elucidated. Therefore, to validate these physiological observations mechanistically, future research must move beyond growth and mineral analysis. Specifically, subsequent studies should focus on quantifying hormonal profiles (particularly auxin and cytokinin) and analyzing the gene expression of root ion transporters to decipher how scion-perceived spectral signals regulate rootstock activity at the molecular level.

## Methods

### Plant source and experimental conditions

The scion and rootstock seedlings were grown in a research glass greenhouse located at the College of Agriculture and Natural Resources, University of Tehran, Karaj, Iran (35°48’ N, 50°59’ E), under controlled environmental conditions: 25/20 °C (day/night), 70% (± 5%) relative humidity, and a natural photoperiod of approximately 12 h (without supplemental lighting). The hybrid tomato (*Solanum lycopersicum* L.) cv. “DRW 7806 F1” (Seminis Vegetable Seeds, St. Louis, MO, USA) was used as the scion, while the interspecific rootstock ‘Maxifort’ (*S. lycopersicum* × *S. habrochaites*) (De Ruiter, United Kingdom) served as the rootstock. All plant experiments were conducted in compliance with relevant institutional, national, and international guidelines and legislation. Seeds of both the rootstock and scion were placed in 72-cell plug trays using a growing medium made of cocopeat and perlite (in a 3:1 ratio, v/v). For nutrition, a seedling-specific fertigation solution was applied, characterized by an EC of 1.8 dS m^-1^ and a pH of 5.8. Irrigation was managed to keep the substrate near its maximum water-holding capacity, resulting in about 30% leachate after each fertigation. The elemental composition of the nutrient solution is detailed in Table 2.

**Table 2:** Specifications of the fertigation solution’s macro- and micronutrient content.

Macronutrients		Micronutrients	
Nutrient	Target concentration (mg/L)	Nutrient	Target concentration (mg/L)
Nitrogen	70	Iron	3
Phosphorus	40	Boron	0.5
Potassium	120	Manganese	0.3
Calcium	100	Zinc	0.3
Magnesium	30	Copper	0.05
		Molybdenum	0.05

### Grafting procedure and healing conditions

Grafting was performed using the splice grafting method when the seedlings reached a stem diameter of 2–3 mm. The scion hypocotyl was cut at a 45° angle 2 cm below the cotyledonary leaves, and the rootstock stem was cut at a matching angle. The cut surfaces were immediately joined and secured with a silicone grafting clip to ensure tight vascular contact. Immediately after grafting, the seedlings were transferred to a specialized healing chamber designed to support the delicate physiological transition at the graft union. From a physiological perspective, the primary objective during this phase was to minimize scion transpiration and maintain cell turgidity while re-establishing functional xylem and phloem connections. To achieve this, seedlings were kept in complete darkness for the first three days. This absence of light was crucial for inducing stomatal closure and halting photosynthetic activity, thereby directing metabolic energy solely toward callus proliferation and tissue regeneration at the graft interface. The chamber was equipped with an ultrasonic humidifier (Model 1200, Iran) controlled by a high-precision sensor (Model AFTF-SD-U, S + S Regeltechnik, Germany) to maintain a relative humidity (RH) of 98 ± 2%. This near-saturation humidity prevented hydraulic failure in the scion, which had been severed from its root system. Temperatures were strictly regulated at 25 °C (day) and 20 °C (night) using a central heating system to favor enzymatic activities related to cell division. After the initial 3-day critical period, representing the onset of callus bridge formation, the RH was gradually reduced to 70% over the next 7 days. This humidity level was maintained constant until the end of the experiment.

### Experimental design and light treatments

The experiment was arranged as a factorial design based on a randomized complete block design (RCBD) with three replicates. The experimental factors included grafting (two levels: grafted and non-grafted) and light spectrum (four levels). Each experimental unit consisted of 10 seedlings. The timeline was as follows: Grafted seedlings first underwent a 3-day dark healing period. Immediately following this phase, both grafted and non-grafted seedlings were placed in climate-controlled chambers with a 16-h photoperiod and exposed to the specific light treatments for 30 days. At that point, they reached the fully developed seedling stage (7–9 true leaves), ready for transplanting. Non-grafted plants were maintained under identical environmental conditions throughout this period to ensure comparability. The illumination provided a photosynthetic photon flux density (PPFD) of 75 ± 5 µmol m^−2^ s^−1^, supplied by 18 W LED wall-washer fixtures (Iran Grow Light Co., Tehran, Iran). The spectral distribution and intensities were measured using a portable spectrometer (SpectroMaster C-7000, Sekonic Co., Japan). Measurements were performed on all seedlings immediately after the 30-day light treatment period. Four distinct light spectra were provided using LED modules with peak wavelengths at 450 ± 10 nm (blue) and 660 ± 10 nm (red). The control group received White (W) light, composed of 49% green, 35% blue, and 16% red, with negligible far-red emission (< 1%) and an estimated phytochrome photostationary state (PPS) of approximately 0.85. The other treatments included: (i) 100% Red (R) with an estimated PPS of 0.89; (ii) 100% Blue (B) with an estimated PPS of 0.49; and (iii) a combination of 70% Red and 30% Blue (R:B) with an estimated PPS of 0.87^67^.

### Morphological measurements and mineral analysis

Measurements were conducted at the commercial seedling stage, immediately following the completion of the 30-day light treatment. Morphological traits, including stem length, root and total dry mass, and leaf area (measured with a LI-3100 Area Meter), were recorded for seedlings that had reached the transplant-ready size (7–9 true leaves). The dry mass was obtained by weighing the samples after a 48-h drying period at 75 °C in an oven. For mineral composition analysis, the dried whole shoot tissues (comprising both leaves and stems) were ground into a fine powder. The analytical methods used for mineral determination included flame photometry (for K) and complexometric titration (for Ca and Mg)^68^. The Kjeldahl method was used to determine total nitrogen (N), and spectrophotometry at 430 nm was employed for phosphorus^69^. The total content of each mineral element in the shoot was calculated using the previously determined element concentration and the shoot dry mass. The final calculation was carried out using Eq. (1).

1 $$TEC= (EC/100)\times SDM$$

where TEC is the Total Element Content (mg), EC is the Element Concentration (%), and SDM is the Shoot Dry Mass (mg).

### Leaf chlorophyll index (SPAD) measurement

A portable chlorophyll meter (SPAD-502, Konica Minolta Corp., Solna, Sweden) was used to measure leaf chlorophyll content non-invasively. This device provides a SPAD value by measuring the leaf’s light absorption at 650 nm (red) and 940 nm (infrared). Measurements were performed on the third fully expanded mature leaf from the apex of each seedling to ensure consistency. To determine chlorophyll content for each replicate, measurements were taken at three points on each leaf^70^To minimize circadian-related physiological variations, all measurements were conducted on the final day of the experiment (after 30 days of light treatment) between 09:00 and 11:00 AM.

### Data analysis

All statistical analyses were performed using SAS software (Version 9.4). We first examined the data for normality using the Shapiro–Wilk test and for homogeneity of variances using Levene’s test to verify that the ANOVA assumptions were met. We then analyzed the experimental data using analysis of variance (ANOVA). When significant treatment effects were detected, we conducted mean separation using the Least Significant Difference (LSD) test at the *P* ≤ 0.05 significance level. To visualize the comprehensive interaction between light quality and grafting across all measured parameters, a heatmap was generated using Microsoft Excel. Given the heterogeneity of measurement units and scales among the studied traits, raw data were standardized by converting values to Z-scores before visualization. The resulting standardized dataset was visualized using a color gradient, with positive Z-scores indicating values above the mean and negative Z-scores indicating values below the mean.

## Data Availability

The data presented in this study are available on request from the corresponding author.
